# Exploring the underlying mechanism between fear of losing power and knowledge hiding

**DOI:** 10.3389/fpsyg.2022.1069012

**Published:** 2022-12-15

**Authors:** Omer Iqbal, Zeeshan Ali, Akbar Azam

**Affiliations:** FAST School of Management, National University of Computer and Emerging Sciences, Lahore, Pakistan

**Keywords:** fear of losing power, knowledge hiding, self-serving behavior, personal competitiveness, moderated mediation

## Abstract

**Introduction:**

Drawing on the assumptions of approach/inhibition theory of power and conservation of resource of theory, this study aims to empirically explore the relationship between fear of losing power and knowledge hiding. To explicate the relationship, this study examines the mediating role of self-serving behavior and moderating role of personal competitiveness.

**Methods:**

To evaluate the relationships, a moderated-mediation model is devised and tested. Data is collected through a web-based questionnaire from 194 individuals employed in both manufacturing and service sector firms of Pakistan. Multiple statistical software packages are used to analyze the data.

**Results:**

After employing several statistical techniques, the findings of the study suggest that self-serving behavior fully mediates the link between fear of losing power and knowledge hiding. Moreover, the result of two-way interaction reveals that personal competitiveness further amplifies the indirect relationship between fear of losing power and knowledge hiding through self-serving behavior.

**Discussion:**

The present study is one of those few types that investigates and uncovers the hidden links between fear of losing power and knowledge hiding. Lastly, theoretical, and practical implications along with future research directions are discussed.

## 1 Introduction

Knowledge hiding (KH) has received due attention in recent times by the scholars of knowledge management (Gagné et al., 2019; Koon, 2022). KH is defined as an “intentional attempt to withhold or conceal knowledge that has been requested by another person” (Connelly et al., 2012). It is relatively a recently emerged phenomenon (Tang et al., 2015) which is very much prevalent in today’s work settings (Semerci, 2019). For example, the findings of a survey indicated that 76% of employees in the USA had been involved in KH during their professional career (Connelly et al., 2012). According to another survey report, 46% of organizational employees in China confessed that they had been involved once in KH (Peng, 2013). KH threatens both organizational and individual performance (Černe et al., 2017; Wang et al., 2018). Moreover, its harmful impact within the organizations are also empirically proven (Bogilović et al., 2017; Bari et al., 2019). For instance, the results of a survey revealed that “Fortune 500” organizations have faced around $31.5 bn annual financial loss when their employees were involved in KH (Babcock, 2004). Similarly, the prevalence of KH behavior have also costed $47 million in productivity of US based organizations in a single year (Panopto, 2018). Panopto (2018) also mentioned that employees in these organizations wasted approximately 5.30 h every week because they had to wait for their colleagues to share the existing knowledge. That wasted time slowed down the overall organizational productivity which turned into a huge financial loss (Kwahk and Park, 2016; Hickland et al., 2020).

Past studies highlighted numerous individual factors that facilitate KH behaviors within the organizations, such as job security (Serenko and Bontis, 2016), territoriality (Huo et al., 2017; Singh, 2019), ostracism at workplace (Zhao et al., 2016), perceived organizational politics (Malik et al., 2019), and injustice (Jahanzeb et al., 2020). In similar vein, power dynamics also play a significant but complex role in affecting individuals’ participation in KH (Issac et al., 2022). Keltner et al. (2003) defined power as “an individual’s relative capacity to modify others’ states by providing or withholding resources or administering punishments”. These resources may be either material (economic) or social (knowledge, affection) and punishments can be both material (physical harm, job termination) or social (ostracism, verbal abuse) (Keltner et al., 2003). The knowledge-power relationship is evident and has explicitly been studied in the past. For instance, knowledge may have a greater potential of fetching power, status, and success for those individuals who own it (Foucault, 1980; Townley, 1993). Hence, if knowledge becomes a source of power, then the owners of such power may have a fear of losing it because they feel that other people may also come to know what they know (Davenport and Prusak, 1998). Kyi (1992) rightly said “It is not power that corrupts but fear. Fear of losing power corrupts those who wield it and fear of the scourge of power corrupts those who are subject to it.” So, when people are uncertain about the future of their organizations, they may attempt to increase their value by enhancing the power which is based solely on their own relevant knowledge (Hinkin and Schriesheim, 1989; Raven, 2008). Considering the knowledge as a source of power, we expect that people having power not only control existing resources but also are involved in such deliberate actions to secure future benefits.

Conversation of resource (COR) theory also indicate that “individuals’ behaviors may be influenced by gain or loss of their own resources” (Hobfoll, 1989; Halbesleben et al., 2014). So, they deliberately hide knowledge to either avoid resource loss or to maximize existing resource (He et al., 2020). From the perspective of COR theory, resources are referred as “those personal characteristics, conditions, objects or energies that are either valued by individuals or serve as a means of acquiring these personal characteristics, conditions, objects, or energies.” These resources also drive individuals in maintaining their existing resources or pursuing new resources (Hobfoll et al., 2000). Thereby individuals are usually hesitant to share knowledge with others because they feel that it has become their competitive advantage and source of power (Bartol et al., 2009). Therefore, based on the stated literary arguments we propose that fear of losing power (FOLP) might be another crucial facet that invokes KH among individuals. The authors are of the belief that this unexplored lens will definitely contribute to the extant literature of KH.

Though power holders have full access to several available resources/benefits, but the FOLP may shift their focus to self-beneficial goals. Similarly, when employees foresee such prospects or situations where their power position is threatened, they usually react negatively and may get involved in unethical conduct such as self-serving behavior (SSB) because they fear losing access to their existing and future benefits and resources (Deng et al., 2018). Thus, this empirical study further examines the potential mediating role of employees’ SSB between FOLP-KH relationship. SSB entails that “individuals disregard group or subordinate interests and, instead, prioritize their self-interest, for instance, by divesting scarce organizational resources away from collective purposes and toward themselves” (Rus et al., 2012). Thus, drawing the on assumptions of approach/inhibition theory of power (AITP) (Keltner et al., 2003), we expect that FOLP may increase employees’ SSB (Wisse et al., 2019) that leads to KH. In line with COR theory, expected loss in power of employees is strongly linked with reduced knowledge sharing as well as increased KH, and these negative practices are executed for more resource (e.g., power) acquisition (Issac et al., 2022).

Alongside behavioral and situational elements, this study further considers the influential moderating role of personal competitiveness (PC), an individual’s characteristic, that further helps to determine KH behavior at the workplace (Hernaus et al., 2018; Nadeem et al., 2020; Fauzi, 2022). PC is defined as “the enjoyment of interpersonal competition and the desire to win and be better than others” (Spence and Helmreich, 1983). Hence, in order to maintain their competitive advantage, individuals are more inclined toward KH so as to keep their power or unique skills intact (Nguyen et al., 2022). Moreover, PC fortifies the tendency of individuals to act self-servingly and engage in less cooperative and more opportunistic behavior (Huo et al., 2017) such as KH. Therefore, it is reasonable to say that individuals who exhibit SSB are more prone to hide knowledge when they are highly competitive in nature than their lesser counterparts.

Connelly et al. (2019) suggested that extensive research is required considering power dynamics that may significantly contribute to KH. Fear is a powerful phenomenon that has the capability to alter individual behaviors. In the same vein, power is also proved to affect individual behaviors. Hence, we expect that fear when combined with power can serve as an antecedent of KH and can further explain the variance in KH behavior. Thus, responding to such need and recent calls in the literature, this study holds some valuable contributions. First, to the best of authors’ knowledge, very less pertinent literature is available that specifically views the association between FOLP and KH. Though Issac et al. (2022) studied the effects of power dynamics (expert power and reward power) with KH in recent times, its relationship with FOLP is still an unexplored avenue and requires due attention which is believed to be a significant theoretical contribution in this domain. Second, we also expect that employees’ SSB has a catalytic mediating role between FOLP-KH relationship. Third, studies confirmed that PC has been a strong predictor in shaping employees’ behaviors about self-interest and KH (Fletcher and Nusbaum, 2010; Hernaus et al., 2018; Semerci, 2019). Hence, it may also be used as a significant element in evaluating the relationship between employees’ SSB and KH.

In sum, we contend that FOLP may have a potency to influence individuals’ intentions to hide knowledge at their workplaces. Specifically, using the tenets of both AITP and COR theory, the underlying mediating role of employees’ SSB and moderating role of PC may also be reckoned while examining the impact of FOLP on KH. So, to achieve the overarching purpose of the current study, we address the following questions:

(1) Does the FOLP positively influence employees’ SSB? (2) Does employees’ SSB positively affect KH? (3) Does employees’ SSB mediates the relationship between FOLP and KH? (4) Does PC moderate the SSB-KH relationship?

**FIGURE 1:**
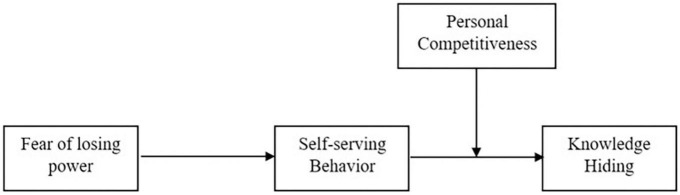
Theoretical framework. Independent variable, Fear of losing power; dependent variable, knowledge hiding; mediator, self-serving behavior; moderator, personal competitiveness, solid lines representing hypothesized relationships.

Figure 1 shows the theoretical framework and relationships to be analyzed.

## 2 Review of literature and hypotheses development

Knowledge is referred to as “the idea, information, or expertise required by the employees of an organization to complete specific tasks” (Alavi and Leidner, 2001; Bartol and Srivastava, 2002; Connelly et al., 2012). Based on the task-related knowledge, employees experience sense of power that propels them to bargain with organizations (Peng, 2013). In knowledge-intensive firms, individuals work in a complex paradigm of power/knowledge that shapes their KH behaviors (Heizmann and Olsson, 2015).

### 2.1 Knowledge hiding

For the organizations to work more effectively and efficiently, many employers expect its employees to freely share their knowledge to others (Connelly et al., 2019). Nevertheless, organizations are dependent on the intellectual assets of its employees (Kelloway and Barling, 2000), and several employees prefer not to share knowledge with others. Though employees have certain reasons of hiding knowledge at the workplace for example, workplace incivility and cynicism (Anand et al., 2022), interpersonal injustice (Cao, 2022), workplace bullying (Islam and Chaudhary, 2022), and peer abusive supervision (Ma et al., 2022) but KH also has some devastating consequences on both individuals and organizations (Serenko and Bontis, 2016; Ellmer and Reichel, 2021; Nguyen et al., 2022). For instance, KH decreases creativity (Černe et al., 2014; Bari et al., 2019), kills innovative work behavior (Černe et al., 2017; Mubarak et al., 2022), and reduces employee performance (Anand and Hassan, 2019). Moreover, it also damages the interpersonal relationship between employees (Connelly and Zweig, 2015) and creates intra-group conflicts (Peng et al., 2020) within the organizations.

Knowledge hiding is an intentional concealment of any information or knowledge from any other individual who has requested it (Serenko and Bontis, 2016). There are different dimensions of KH prevailing in the organizations but in literature, three main dimensions of KH have firstly been identified and described extensively by Connelly et al. (2012), these include: (1) evasive hiding, which involves the behavior of individuals providing incorrect or incomplete information that may not yield the desired outcome, (2) rationalized hiding, in which individuals hide any information or knowledge but offers a clear explanation and logic of hiding it, and (3) playing dumb, in which individuals pretend to know nothing about the knowledge which is requested (Connelly et al., 2012; Connelly and Zweig, 2015). Organizational employees may involve is such activities for a number of reasons, including fear of losing influence (power), position or wealth (Cao, 2022). Since each dimension holds a negative connotation (Ghani et al., 2020), this study examines all dimensions of KH as a single construct.

### 2.2 The knowledge-power nexus

There are various theoretical perspectives focusing on how having power influences individuals and their behaviors at workplaces. For instance, one of the aspects of AITP states that “reduced power is linked with increased threat and punishment and social constraint, and should thereby activate inhibition-related effect, cognition, and behavior” (Keltner et al., 2003). Some individuals follow “knowledge is power” dictum in organizations and become knowledge hiders as they do not want to lose their power position by sharing knowledge with others (William, 2008). Similarly, individuals intentionally hide knowledge with their co-workers when seeking power positions (Anand and Hassan, 2019). For instance, an employee may have a fear of losing his/her unique status or power by sharing some knowledge (Cress et al., 2005). Thus, by reviewing several studies divulging knowledge-power nexus within the organizational context, it is argued that FOLP may compel employees to conceal or withhold knowledge with others to have protection against their potential replacements. Because, if knowledge contributes as an element of retaining or gaining more power, then KH may become a rational strategy (Ferraris et al., 2018). The purpose behind KH by individuals is to remain dominant and indispensable within the organization (Butt, 2021). Expected loss in power of employees is strongly linked with reduced knowledge sharing as well as increased KH, and these negative practices are executed for more power acquisition (Issac et al., 2022).

Embedded in AITP (Keltner et al., 2003), the literature of social psychology discussed various aspects of power. Powerful individuals not only have the control over resources (Fiske et al., 2010) but also have the “ability to affect others while remaining immune from influence” (Galinsky et al., 2008). These concepts imply that power lies in organizational structures whereby employees have the opportunities to influence others and possess resources (Luqman et al., 2022). However, Galinsky et al. (2003) argue that power is also a psychological state that varies by the situation at any point of time in the workplace. For individuals, psychological power can be satisfying and pleasant as it increases the sense of accomplishment (Wang et al., 2019) or may cause stress that carries some dangers, for instance, promoting envy, abuse, and dislike at the workplace (Yu et al., 2018). In relation to this, individuals with unstable power positions exhibit higher levels of stress than others (Jordan et al., 2011). Thus, we believe that fear is a stressor that depletes individuals’ resource (i.e., power) and stimulate their SSB. The COR theory (Hobfoll, 1989) also argues that when individuals are posed to fear in losing their resource, it cause stress. Consequently, they attempt to regain their lost resources by engaging in different behaviors (Fatima et al., 2020).

### 2.3 Mediation of self-serving behavior

At one time Napoleon Bonaparte said, “Power is my mistress. I have worked too hard at her conquest to allow anyone to take away from me.” This quotation rightly exemplifies that individuals who are on power positions, always want to hold them in any case (Fehr et al., 2013; Saguy and Kteily, 2014). Indeed, empirical research has proven that power-holder usually tends to reinforce and maintain his/her superior position within the organization (Anderson and Brion, 2014). The AITP (Keltner et al., 2003) proposes that power has a broad-ranging behavioral and psychological consequences that shape employees’ perception about themselves, and the people around them. In addition, empirical findings also suggest that individuals’ power may in fact affect both self and group behaviors (Keltner et al., 2003). The AITP provides a very useful lens to understand the experiences of individuals, when low in power, are likely to perceive uncertainties and threats, thus exhibit negative behaviors at the workplace (Berdahl and Martorana, 2006). For instance, studies proved that less powerful individuals are more susceptible to exhibit SSB (Chen et al., 2001; Anderson et al., 2003; Guinote, 2007), and act in more invariable ways than high power counterparts (Guinote et al., 2002). Furthermore, individuals at the workplace, when they feel threatened to lose their power, usually resort to SSB (Georgesen and Harris, 2006).

From the preceding literature, it is evident that power as resource may be a strong predictor of shaping individuals’ behaviors at the workplace. And fear of losing such resources may motivate employees to indulge in more SSB. Because the depletion in resources is related to destructive behavior (Mohsin et al., 2022). Hence, it prompts employees to conserve their resources (Holmgreen et al., 2017). Further, employees who engage in SSB may use their positions to get either benefits for themselves at the cost of their co-worker, shift blame of their own faults onto their subordinates, or get credit claims for tasks done by their subordinates (Wisse et al., 2019). Thus, we expect that FOLP strongly intensifies employees’ unethical actions such as SSB at the workplace. This is hypothesized as below:

*H1: Fear of losing power has a significant and positive impact on employees’ self-serving behavior.*

Decoster et al. (2014) argued that employees’ SSB has many detrimental effects on workplaces. These include, cheating, corruption, stealing, loafing, and KH (Peng et al., 2019). The strategy of KH enables employees to not only maximizing their personal interest but gaining competitive advantage at the workplace (Siachou et al., 2021). To get personal benefits and more incentives at the workplace, employees also misguide their colleagues, thus their intention in involving KH becomes justified (Sulistiawan et al., 2022). The COR theory also proposes that anticipated loss or gains of resources are significant contributors in determining employees’ behavior (Hobfoll, 1989). In this study, we are focusing on KH as an outcome of SSB that not only restricts employees from generating creative ideas at the workplace but also predicts a reciprocal loop of distrust in which employees hesitate in sharing knowledge with others (Černe et al., 2014) in order to safeguard their personal interests (Connelly et al., 2012). Therefore, the following hypothesis is devised:

*H2: Employees’ self-serving behavior has a positive relationship with knowledge hiding.*

Based on the pertinent literature, it may be reasoned that individuals who acquire knowledge after investing a handsome amount of effort and time, may have a strong ownership, and power over their knowledge, which urge themselves to behave self-servingly by not sharing that knowledge to avoid their personal loss, that subsequently triggers their KH behavior. Therefore, we hypothesized is as below:

*H3: The self-serving behavior of employees positively mediates the relationship between fear of losing power and knowledge hiding.*

### 2.4 The moderating role of personal competitiveness

Personal competitiveness may be a characteristic of an individual that varies across people due to their individual differences (Fletcher et al., 2008). It is conceived as “individuals who enjoy interpersonal competition and are motivated to win or do better than others” (Spence and Helmreich, 1983; Schneider et al., 2005). This statement is coherent with the idea of Kohn (1992) about intentional competitiveness which is “the desire on the part of the individual to be number one” (Kohn, 1992). A high level of competitiveness urges individuals to contend and want to succeed at any cost to enhance or maintain their beliefs of self-worth (Horney, 1937). Although FOLP may foster SSB leading individuals to KH, yet we expect that the effect may vary due to different personality traits of individuals. In this study, we propose that PC is one of those traits that significantly influence employees’ behaviors at the workplace. Studies confirmed that competitiveness at the workplace not only adds to employees’ perception about competition, but affects their behavior and attitude as well (Fletcher and Nusbaum, 2010; Jha and Varkkey, 2018). The perception of high competitiveness among individuals may also lead to uncertainty, interpersonal conflict, and stress among employees (Semerci, 2019) that result in undesirable organizational consequences (i.e., self-serving, counterproductive/deviant behaviors) (Hernaus et al., 2018). Moreover, competitive individuals attempt to maximize their personal interests for seeking individual status, rewards and recognition (Van Lange et al., 1997). In a similar vein, competitive individuals are usually goal-driven who want to win at any cost (Brown et al., 1998). Thus, in line with the COR theory, individuals strive to create a surplus of resource (e.g., power) by reinvesting their existing resources (Hobfoll et al., 2000).

Connelly et al. (2014) reported that PC predicts perceived competition and prompted individuals to hide knowledge. Thus, PC may indirectly lead employees feeling “too busy” to share their knowledge when it is requested (Connelly et al., 2014). In addition, Hernaus et al. (2018) indicated that organizational employees would be more likely to hide knowledge when they are highly competitive. Sharing knowledge at the workplace may threaten personal interest or even harm PC of those individuals who share it (Connelly et al., 2012). Building on preceding literature, we expect that competitive individuals, driven by self-interest, are more susceptible to hiding knowledge at their workplaces. Further, the relationship of SSB-KH will be stronger for those individuals who have a high degree of PC. Moreover, Therefore, it is hypothesized as below:

*H4: Personal competitiveness will positively moderate the relationship between self-serving behavior of employees and knowledge hiding. However, this positive relationship will be stronger (weaker) at higher (lower) levels of personal competitiveness.*

In general, a competitive working climate prompt individuals for a constant competition among each other to get a share from the organizational resources which are limited in number (Wayne and Ferris, 1990). Thus, the individuals who are highly competitive in nature usually have greater chance to earn a power position. However, the stakes of losing power are equally higher than in a less competitive climate (Wisse et al., 2019). COR theory proposes individuals’ reactions to resource depletion is contingent on their traits (Hobfoll et al., 2000). So, individuals having competitiveness as a trait may behave negatively when expected in their resource loss. Research found that FOLP may be highly stressful and imperiling for the individuals (Jordan et al., 2011), subsequently it evokes less cooperative and more opportunistic behavior (i.e., KH) especially among competitive individuals. From the lens of COR theory, individuals’ reaction to resource loss, related to workplace stressor, is dependent on individual difference (Hobfoll et al., 2000). Hence, it is fair to argue that individuals’ inclination to serve themselves, embedded in FOLP, will lead them to exhibit KH behavior, especially when they are competitive. Therefore, it comes up as:

*H5: Personal competitiveness will positively moderate the indirect relationship of fear of losing power on knowledge hiding via self-serving behavior. However, this indirect relationship will be stronger (weaker) at higher (lower) level of personal competitiveness.*

## 3 Methodology and methods

### 3.1 Research approach

All devised hypotheses were validated using the design of quantitative research along with a deductive approach. Specifically, a survey-based non-experimental research design was adopted to conduct the study.

### 3.2 Procedure

For this empirical study, data was gathered using a web-based survey questionnaire which was distributed to the individuals employed in service and manufacturing sector of Pakistan. The questionnaire was formulated using English language as it is being considered as the official language in all sector organizations of Pakistan. Previously, survey research conducted in Pakistan also used English language in questionnaires (Khan et al., 2015; Raja et al., 2018; Khattak et al., 2020). Since, this study involved human participants, additional information about maintaining participants’ confidentiality was added in the prefatory section of the questionnaire so to have fair responses. To make a proximal separation, entire data were gathered in two-waves to minimize the issue of common method bias (Podsakoff et al., 2012). In the first wave (at Time 1), 220 respondents were asked about their opinions on FOLP and PC. Moreover, they were also asked to share their demographics (e.g., age, gender, work experience, education etc.) and rate questions on social desirability bias (SDB) (Crowne and Marlowe, 1960).

### 3.3 Sample

All the respondents were selected through a convenience-based sampling technique from both service and manufacturing sector firms as it contained a broad range of knowledge-intensive firms in Pakistan such as academia, food, packaging, healthcare, telecom, IT, and banks. This sampling technique helped the researchers to gather data from the respondents in a cost-effective manner (Nawaz et al., 2020; Yingfei et al., 2022). In addition, the selection of the firms was done using personal and professional links; contacts of one of the authors who also assisted in data collection. Out of 220 randomly selected employees, 209 completed the survey (response rate 95%). Four weeks later, at time 2, these 209 employees were approached again and asked to rate their tendencies about exhibiting self-serving and KH behaviors. After equating the data, the final sample of 194 was found reasonable and valid with an overall 88% response rate. The timing of both surveys was set up in a way to establish the logic that FOLP would predict SSB that in turn would be related to KH. Of the 194 respondents, 131 (64.52%) were male and 63 (32.45%) females who participated in the survey research. In terms of level of education, 2.1% were having secondary school certificates, 3.6% had a college degree, 27.3% were university graduates, 62.9% had post graduate university degrees, and 4.1% were having doctoral degrees in their accounts. The average age of the respondents was 34.49 years (SD = 6.53), and the mean work experience was 11.03 years (SD = 5.98).

### 3.4 Measures

In this study, a Five-point Likert scale ranging from 1 (“Strongly Disagree”) to 5 (“Strongly Agree”) was used to measure the gathered responses of all study variables. Moreover, the reliability of each variable was compared and assessed against the Cronbach’s Alpha value i.e., 0.7 or higher (Sarstedt et al., 2019).

#### 3.4.1 Independent variable: Fear of losing power

Fear of losing power; was assessed by employing a three-item scale based on the study by Wisse et al. (2019). All three items of this scale *included “I sometimes fear that my position will be undermined by my colleagues”, “I sometimes feel that some of my colleagues are striving for my position”*, and *“I am sometimes apprehensive (feeling) about my colleagues resisting my directives”* The coefficient of Cronbach (α) for FOLP was 0.72; thus, this variable found reliable.

#### 3.4.2 Dependent variable: Knowledge hiding

Knowledge hiding was taken as a single construct (including all three forms e.g., rationalized hiding, evasive hiding, and playing dumb) and gauged on 12-item scale devised by Connelly et al. (2012) This scale covered three sub-dimension of knowledge hiding i.e., rationalized hiding, evasive hiding, and laying dumb, comprising four items each. One of the scale items of each dimension included, *“I agree to help him/her but never really intend to”;* “*I pretend that I do not know the information*”; and *“I explain that I would like to tell him/her but was not supposed to.”* The Cronbach’s alpha (α) value for KH was 0.89 and found reliable.

#### 3.4.3 Mediating variable: Self-serving behavior

Self-serving behavior was evaluated by adapting an eight-item scale devised by Rus et al. (2010). This scale opened with the following sample item: *“I have negotiated a bonus for myself that was substantially higher than the bonus my subordinates received.”* The Cronbach’s alpha (α) value for SSB was 0.75; therefore, this variable was reliable.

#### 3.4.4 Moderating variable: Personal competitiveness

To gauge PC, a 10-item scale formulated by Lu et al. (2013) was adapted for this particular study. This scale covered three sub-dimensions of PC i.e., behavioral tendencies of competition, three items; beliefs about competition, three items; and feeling for competition, four items. One of the sample items of each sub-dimension was written as *“Even in a group working toward a common goal, I still want to outperform others”, “I like competition because that it gives me a chance to discover my own potential”*, and *“Being outperformed by other members in the group annoys me”.* The coefficient of Cronbach (α) for PC was 0.81; hence, the value was found reliable.

#### 3.4.5 Control variables

This study includes age, gender, work experience, education, and SDB as control variables because previous research suggest that these variables may cause a potential confounding effect on human behaviors and are directly related to the main study variables (Marcus and Schuler, 2004; Kish-Gephart et al., 2010; Wang and Noe, 2010).

## 4 Analysis of data and results

### 4.1 Validity analyses and measurement of model

We conducted a series of confirmatory factor analyses (CFAs) using JASP 0.14.1 to examine the discriminant validity between study variables. Model fitness was analyzed by on the bases of fit indices i.e., RMSEA, SRMR, TLI, and CFI. The discriminant validity of study variables emerged by comparing the fit of the constrained models. As shown in Table 1, the hypothesized four-factor model (fear of losing power, self-serving behavior, personal competitiveness, and knowledge hiding) yielded a better fit within the available data i.e., χ^2^/df = 1.85, RMSEA = 0.066, SRMR = 0.062, TLI = 0.91, and CFI = 0.94 than alternative series of three-, two-, and one-factor models which indicate adequate discriminant validity. All of these indicators were above the cut off values as suggested by Hu and Bentler (1999). Moreover, all related items significantly loaded on their associated factors, hence confirming convergent validity of all study variables. These resulting values provided enough support to establish construct validity of all study variables.

**TABLE 1:** Confirmatory factor analysis.

Description	χ^2^	df	χ^2^/df	RMSEA	SRMR	TLI	CFI
One-factor model	316.38	54.00	5.86	0.158	0.135	0.49	0.58
Two-factor model	201.40	53.00	3.80	0.120	0.104	0.71	0.77
Three-factor model	162.80	51.00	3.19	0.106	0.092	0.77	0.82
Four-factor model	88.67	48.00	1.85	0.066	0.062	0.91	0.94

One-factor model: (FOLP, SSB, PC, KH combined); two-factor model: FOLP, (SSB, PC, KH combined); three-factor model: FOLP, (SSB, PC combined), KH; four-factor model: FOLP. SSB, POP, KH. FOLP, fear of losing power; SSB, self-serving behavior; PC, personal competitiveness; KH, knowledge hiding; RMSEA, root mean square error of approximation; SRMR, standardized root mean square; TLI, tucker-lewis index; CFI, comparative fit index.

### 4.2 Descriptive statistics and correlation analysis

The descriptive statistics (means, standard deviations) and correlation analysis (Pearson’s *r*) of the study variables are reported in Table 2.

**TABLE 2:** Descriptive statistics and correlation analysis.

	Mean	SD	1	2	3	4	5	6	7	8	9
1. Gender	0.67	0.47	—	—	—	—	—	—	—	—	—
2. Age	34.49	6.52	0.15*	—		—	—	—	—	—	—
3. W/Exp	11.03	5.98	0.20**	0.91**	—	—	—	—	—	—	—
4. Edu	3.63	0.72	−0.24**	0.07	–0.03	—	—	—	—	—	—
5. SDB	3.38	0.50	0.12	0.08	0.09	0.08	—	—	—	—	—
6. FOLP	3.16	0.98	–0.02	–0.02	–0.05	0.05	–0.01	—	—	—	—
7. SSB	2.08	0.63	0.21**	0.03	0.03	–0.13	–0.14	0.22**	—	—	—
8. PC	3.09	0.63	–0.06	–0.04	–0.01	0.01	–0.13	0.10	0.10	—	—
9. KH	2.15	0.74	0.17*	0.05	0.07	–0.06	–0.06	0.05	0.54***	0.13	—

Gender, (0 = female; 1 = male); age and work experience are coded as continuous variables. W/Exp, work experience; SDB, social desirability bias; FOLP, fear of losing power; SSB, self-serving behavior; PC, personal competitiveness; KH, knowledge hiding. Significance Levels: *p < 0.05, **p < 0.01, ***p < 0.001.

### 4.3 Hypotheses testing

The hypothesized four-factor model (Figure 1) represents a moderated-mediation model in which the moderating effect is lying at the stage 2 of the mediation pathway. We employed a series of hierarchical regression models using PROCESS macro v3.5 (Hayes, 2017) in SPSS 24.0 to test hypothesized model including direct, indirect and conditional indirect effects. Specifically, while estimating the conditional indirect effect(s), both SSB and PC were mean centered (Agerström et al., 2005) prior to calculate the interactional effect and to avert multicollinearity (Aiken et al., 1991). Furthermore, to analyze the effects of mediation, moderation, and moderated mediation, a bootstrapped approach including 5,000 samples at 95% confidence interval (CI) was employed (Preacher et al., 2007). The indirect effects stand significant and valid if the CIs are without zero value (Hayes, 2013). The PROCESS macro v3.5 also helped in avoiding statistical issues of power that may come from abnormal or asymmetric sampling distributions of indirect relationships (MacKinnon et al., 2004; Hayes, 2013).

Table 3 reports the results of all regression models run to test the hypotheses. It showed that FOLP had a positive and significant impact on employees’ SSB, hence provided full support to H1. These results also showed a significantly positive relationship between employees’ SSB and KH, thus supported H2. Similarly, the resulting values of indirect effect (i.e., mediation) also indicated that CIs were without zero value (i.e., β = 0.09, SE = 0.03, CI = 0.02, 0.17), hence lend ample support to H3 (i.e., full mediation of employees’ SSB between FOLP-KH relationship).

**TABLE 3:** Regression models and results.

	SSB				KH			
	Model 1				Model 2			
Variable	β	SE	t	LLCI, ULCI	β	SE	t	LLCI, ULCI
**Control variables**								
Gender	0.28**	0.09	2.97	0.09, 0.47	0.07	0.10	0.75	–0.12, 0.28
Age	0.01	0.01	0.44	–0.02, 0.04	–0.01	0.01	–0.73	–0.04, 0.02
Work experience	–0.01	0.01	–0.31	–0.04, 0.03	0.01	0.02	0.99	–0.01, 0.05
Education	–0.07	0.06	–1.19	–0.02, 0.05	0.03	0.06	0.51	–0.09, 0.16
SDB	–0.19*	0.08	–2.24	–0.36, –0.02	0.01	0.09	0.20	–0.16, 0.20
**Study variables**								
FOLP	0.14**	0.04	3.29	0.05, 0.23	–0.03	0.04	–0.78	–0.13, 0.05
SSB	–	–	–	–	0.60^***^	0.07	7.70	0.44, 0.75
PC	–	–	–	–	0.10	0.07	1.43	–0.04, 0.24
SSB × PC	–	–	–	–	0.23*	0.10	2.25	0.02, 0.44
R-square	0.13				0.33			
F—statistics	F (6, 187) = 4.64 *p* < 0.001				F (9, 184) = 10.18 *p* < 0.001			

*N* = 194, Gender, (0 = female; 1 = male); age and work experience are coded as continuous variables. *p < 0.05, **p < 0.01, ***p < 0.001. LLCI, lower limit confidence interval; ULCI, upper limit confidence interval; SDB, social desirability bias; FOLP, fear of losing power; SSB, self-serving behavior; PC, personal competitiveness; KH, knowledge hiding.

**FIGURE 2:**
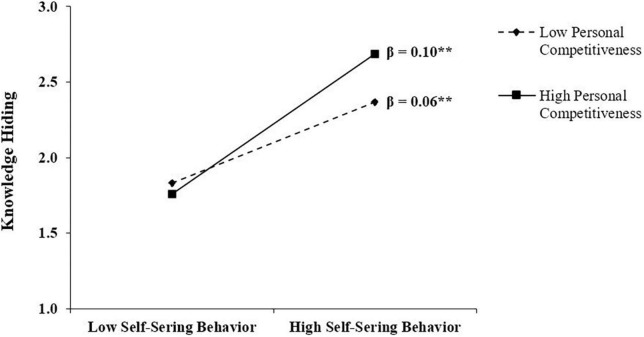
The moderating effect. Figure represents the moderating effect of personal competitiveness on the relationship between employees’ self-serving behavior and knowledge hiding.

In support of H4, the results as shown in Table 3 suggested that PC positively moderated the link between employees’ SSB and KH because the coefficient of two-way interaction (SSB × PC) was found significant and positive (i.e., β = 0.23, SE = 0.10, *p* < 0.05), thus the argument of H4 is supported. This two-way interaction is plotted in Figure 2.

Table 4 reports the results of conditional indirect effect (i.e., FOLP → SSB → KH). It showed that at both values of PC, the effect sizes were different. At low values of PC, the effect of conditional indirect effect was significant and positive (no zero exists between LLCI and ULCI) (i.e., β = 0.06, SE = 0.02, 95% CI = 0.01, 0.13), and became more stronger at high value of PC (i.e., β = 0.10, SE = 0.04, 95% CI = 0.03, 0.20). Hence, H5 is supported. The test results for all hypotheses also supported the conditions of moderated mediation because coefficients of FOLP at first stage, and the interaction (SSB × PC) at the second stage were significant. Moreover, the results of index value of moderated mediation were significant because CI did not include zero, hence evident enough to support a successful moderated mediation at the second stage of mediation pathway (i.e., Index = 0.34, SE = 0.02, 95% CI = 0.00, 0.07).

**TABLE 4:** Conditional indirect effect.

Levels	Conditional indirect effect	Boot SE	Boot LLCI, ULCI
Low PC (–0.63)	0.06	0.02	0.01, 013
Mean (0.00)	0.08	0.35	0.02, 0.16
High PC (0.63)	0.10	0.04	0.03, 0.20

*N* = 194. Bootstrap sample size = 5,000. LLCI, lower limit confidence interval; ULCI, upper limit confidence interval; PC, personal competitiveness.

## 5 Discussion

We drew the AITP (Keltner et al., 2003) and COR theory (Hobfoll et al., 2000) to argue that FOLP may have a potency to positively influence individuals’ intentions to hide knowledge at their workplaces. Therefore, based upon this logic first, we examined the influential positive role of FOLP on employees’ SSB (H1). The findings confirmed our prediction that FOLP has a significant and positive impact on employees’ SSB. These findings are aligned with the existing research showing that unsafe working environment reduces the psychological cognitive resources of power holders that, in turn, induces counterproductive and deviant behaviors (Kahn, 1990; Luqman et al., 2022). Second, we determined that employees’ SSB is positively associated with KH (H2). In support of this argument, recent studies has demonstrated that individuals’ behavioral factors such as attitude, competitiveness, self-interest, privacy, and trust play a crucial role in affecting their intentions to hide knowledge (Nadeem et al., 2020; Fauzi, 2022). Our second contribution unveils the connecting role of employees’ SSB to the link between FOLP and KH (H3). Organizational individuals with less pride emotions are likely to feel unsafe in organizational elements, which allow them to become hubristic (arrogant, self-interested etc.) that elevate their KH behavior (Han et al., 2021). To follow this reasoning, our findings reveal that employees’ SSB does play a pivotal role in FOLP-KH relationship. However, these findings are inconsistent with the argument of Issac et al. (2022) suggesting that an increase in power prompt individuals to exhibit KH behavior at the workplace. Finally, we investigated the moderating role of PC on the relationship between employees’ SSB and KH. The findings supported our prediction and reveal that PC positively moderates the link between employees’ SSB and KH (H4). Moreover, the combined effect of individual characteristics (i.e., SSB and PC) on KH is found significant, hence complemented recent research confirming that individual characteristics are important predictors for KH behavior (Hernaus et al., 2018; Fauzi, 2022). These findings successfully contribute to the literature of personality as we analyzed how some personality traits (i.e., SSB and PC) of employees affect their KH behavior. In sum, the findings of this study validate the proposition that highly competitive self-serving employees, when being afraid losing their power, are more likely to hide knowledge with others at the workplace.

## 6 Implications

### 6.1 Theoretical implications

This study adds to our knowledge by examining the underlying roles between FOLP and KH. First, in existing business world, power dynamics are shifting on regular basis especially in the organizational contexts and experienced employees are no more complying with relatively predictable career paths (Savickas et al., 2009). In such contexts, individuals are more prone to involve in activities such as KH that may harm organizational performance at large. Peng (2013) argued that organizational employees are more likely to exhibit KH behavior when they feel that the knowledge which is used at the workplace, is their personal property and an effective source of gaining personal power. So, fear of losing such power may urge themselves to depict more KH behaviors. Issac et al. (2022) implied that expected shifts in power strongly affect individuals’ decision to share knowledge. To follow this reasoning, the empirical findings of the current study underscore the significant role of FOLP in triggering KH behavior in organizations, thus, furnishing a solid foundation for further inquiry about KH. Second, this study also supported that SSB is one of the key underlying mechanisms that connects FOLP to KH and comes out as a good mediator to uncover the effect of FOLP on KH. In the past, authors have debated extensively on the relationship between power dynamics and SSB. For example, Rus et al. (2012) contended that when employees are not held accountable, they use their power and are more likely to exhibit SSB. Moreover, a few studies also considered the potential adverse outcomes of SSB both at the organizational and individual and levels (Carmeli and Sheaffer, 2009; Peterson et al., 2012). This study proposes that KH may be one of those outcomes of SSB that may negatively affect both individual and organizational performance. For a better realization about the phenomenon of KH, it is important to explore all its antecedents that may shed some light and contribute to the literature of knowledge management (Semerci, 2019). In this study, SSB, strongly influenced by FOLP, appeared as a strong predictor of KH, which the authors believe that it adds value to the pertinent literature. Third, the findings of this study successfully established that highly competitive individuals, driven by self-interest are more likely to exhibit KH behaviors. The consideration of individual characteristics e.g., PC played a fruitful role to a better understanding of the association between SSB and KH. Though individual characteristics have proven to exert a triggering effect on KH (Peng et al., 2020), thus it is believed that results of this study will add value to the literature of psychology, personality, organizational behavior, and KH.

### 6.2 Practical implications

Understanding when and how individuals’ FOLP impacts their KH behavior has some serious practical implications. First, the empirical result of this study opens two broader debatable topics: (1) Organizations should review their policies (HR, compensation, equity etc.) and systems of justice and fairness that are causing fear among individuals losing their power and knowledge. So, for a conducive work environment the organizations may introduce a culture of knowledge sharing, promoting teamwork, adopting different knowledge management tools for advancing employees’ commitment toward their organizations. (2) KH behaviors may be curbed by focusing on management practices such as psychological safety, empowering teams etc. that may reduce employees’ self-perception of retaining knowledge and expertise. Because a sense of insecurity, if not addressed, may cause FOLP and elicit KH behavior among employees. Thus, organizations employers should strongly focus on individuals’ psychological safety at the workplace as it may not only exert negative effect on KH (He et al., 2020), but also help in reducing fears about losing stakes like status, power, or career. Second, this research found that SSB mediates the link between FOLP and KH. Therefore, a culture of trust, and transparency should be developed and promoted to control such behaviors. For example, if organizational employees trust that employers value fair decision-making regarding demotions, or dismissals etc., they may feel fearless and more likely to leave their personal interests rather than group interests (Aquino et al., 2006). Lastly, organizations should also focus on a healthy working environment to curb undesired behaviors such as KH. In a working environment, individuals need to emphasize not only sharing, cooperating, and improving their skills, but also proving themselves to achieve desired results (DeShon and Gillespie, 2005). However, the challenge is to keep and maintain a balance among all whereby employees not only emphasize sensing shared fates, cooperation and learning but also focus on achieving higher performance.

### 6.3 Suggestions for policy makers

Considering the factors leading to KH, it may also be inferred that KH is not the issue related to individuals only; rather it covers all organizational aspects. We propose some remedial measures that policy makers in the organizations need to emphasize on, so as to address the elements that influence KH. First, to overcome the issues that cause fear and insecurities among employees, there is a dire need of inculcating core self-evaluations which include self-efficacy, self-esteem, emotional stability, and loci of control. It helps to motivate employees to perform better in their respective work areas. To implement this effectively, employers should offer multiple training programs to enhance the core self-evaluations of organizational employees. For instance, they may offer either financial or non-financial rewards to recognize their efforts. This will help individuals to overcome their fear and insecurities, which as a result will control their issues of seeking revenge, feeling powerless, or other intentions may cause KH (Anand and Hassan, 2019). Second, employers should also educate its employees about the importance of knowledge sharing. This would help colleagues empathize with each other to perform their tasks efficiently. At the same time, employees should also be aware from the adverse effects of KH and must be well-informed that KH behavior will be discouraged and not to be appreciated. Third, the findings of our study suggest that expected shifts/fears of power loss encourages employees’ KH behavior, such scenarios advocate managers to provide and maintain a workplace that ensures the psychological safety of employees (Tan et al., 2022). This might be done by instilling confidence within employees who possess higher levels of knowledge than others. Thus, employers should get them involved in decision-making, especially in the tasks/activities in which they are expert of. Such initiative will not help limiting their KH behavior but also ameliorate their relevant knowledge, skills, and abilities. Based on this, organizational hierarchies may be restructured reflecting key positions for such individuals, or they may be designated with specialized positions that distinguish them from others.

## 7 Limitations and directions for future research

This empirical study has few limitations. First, the present study considered power dynamics in general, and empirically tested its relationship with KH. However, specified dimensions of power (French et al., 1959) may also be considered to further explore the relevance and underlying mechanisms with other types of undesired behaviors such as KH, and deviant/counterproductive work behaviors etc. Second, this empirical study found employees’ SSB as a strong mediator between FOLP-KH relationship. However, other individual factors such as perceived envy, and felt obligation may also be considered as mediating mechanisms that may further expatiate the status of KH (Liu et al., 2020). Third, this study found that highly competitive individuals are more prone to exhibit KH behaviors. Nevertheless, other individual and organizational elements may also be taken to evaluate the connection between power dynamics and KH. For instance, conscientiousness is one of those personality traits that has a tendency to put effort (Mount and Barrick, 1995) and withhold counterproductive/deviant work behavior such as KH (Sackett and DeVore, 2001). In addition, Islamic work ethics may also be considered for future analyses in curbing negative behaviors like KH at the workplace (Islam et al., 2021).

## 8 Conclusion

The findings of this study help to enrich the extant literature on the detrimental effects of FOLP within the organizational contexts by investigating the catalytic roles of employees’ SSB and PC on KH. Moreover, we illustrate that employees’ SSB positively mediates FOLP-KH relationship. Further, PC also moderates the employees’ SSB and KH relationship. The findings of the two-way interaction between SSB and PC escalate KH behavior of employees. Finally, this study presents the importance of considering both contextual and individual elements while studying KH within organizations.

## Data availability statement

The raw data supporting the conclusions of this article will be made available by the authors, without undue reservation.

## Ethics statement

The studies involving human participants were reviewed and approved by the Committee of Professional Ethics, FAST School of Management, National University of Computer and Emerging Sciences, Lahore Campus. The patients/participants provided their written informed consent to participate in this study. This study was conducted in accordance with the Declaration of Helsinki guidelines.

## Author contributions

OI and ZA: conceptualization. OI: formal analysis and writing—original draft. OI, ZA, and AA: methodology. ZA and AA: project administration and supervision. OI and AA: writing—review and editing. All authors have fully agreed to the published version of this manuscript.
